# Limited evidence of association between dysregulated immune marker levels and telomere length in severe mental disorders

**DOI:** 10.1017/neu.2024.62

**Published:** 2025-01-23

**Authors:** Monica B.E.G. Ormerod, Thor Ueland, Monica Aas, Gabriela Hjell, Linn Rødevand, Linn Sofie Sæther, Synve Hoffart Lunding, Ingrid Torp Johansen, Vid Mlakar, Dimitrios Andreou, Torill Ueland, Trine V. Lagerberg, Ingrid Melle, Srdjan Djurovic, Ole A. Andreassen, Nils Eiel Steen

**Affiliations:** 1 Division of Mental Health and Addiction, Oslo University Hospital, Oslo, Norway; 2 Institute of Clinical Medicine, University of Oslo, Oslo, Norway; 3 Research Institute of Internal Medicine, Oslo University Hospital, Rikshospitalet, Oslo, Norway; 4 Thrombosis Research Center (TREC), Division of Internal Medicine, University Hospital of North Norway, Tromsø, Norway; 5 Department of Psychiatry, Ostfold Hospital, Graalum, Norway; 6 Department of Psychology, University of Oslo, Oslo, Norway; 7 Department of Medical Genetics, Oslo University Hospital and University of Oslo, Oslo, Norway; 8 NORMENT, Department of Clinical Science, University of Bergen, Bergen, Norway; 9 Department of Clinical Neuroscience, Centre for Psychiatry Research, Karolinska Institutet & Stockholm Health Care Services, Stockholm County Council, Stockholm, Sweden; 10 Social, Genetic and Developmental Psychiatry Centre, Institute of Psychiatry, Psychology and Neuroscience, King’s College London, London, UK; 11 Department of Psychosis Studies, Institute of Psychiatry, Psychology & Neuroscience, King’s College London, London, England, UK; 12 Department of Behavioural Sciences, OsloMet – Oslo Metropolitan University, Oslo, Norway; 13 Division of Mental Health and Substance Abuse, Diakonhjemmet Hospital, Oslo, Norway

**Keywords:** Schizophrenia, Bipolar disorder, Cytokines, Telomere, Ageing

## Abstract

**Objective:**

Accelerated ageing indexed by telomere attrition is suggested in schizophrenia spectrum- (SCZ) and bipolar disorders (BD). While inflammation may promote telomere shortening, few studies have investigated the association between telomere length (TL) and markers of immune activation and inflammation in severe mental disorders.

**Methods:**

Leucocyte TL defined as telomere template/amount of single-copy gene template (T/S ratio), was determined in participants with SCZ (*N* = 301) or BD (*N* = 211) and a healthy control group (HC, *N* = 378). TL was analysed with linear regressions for associations with levels of 12 immune markers linked to SCZ or BD. Adjustments were made for a broad range of potential confounding variables. TL was measured by quantitative polymerase chain reaction (qPCR) and the immune markers were measured by enzyme immunoassays.

**Results:**

A positive association between levels of soluble tumour necrosis factor receptor 1A (sTNF-R1) and TL in SCZ (*β* = 0.191, *p* = 0.012) was observed. Plasma levels of the other immune markers were not significantly associated with TL in the BD, SCZ or HC groups.

**Conclusion:**

There was limited evidence of association between immune markers and TL in SCZ and BD. The results provide little support for involvement of immune dysregulation, as reflected by current systemic markers, in telomere attrition-related accelerated ageing in severe mental disorders.

## Graphical abstract

**Figure:**
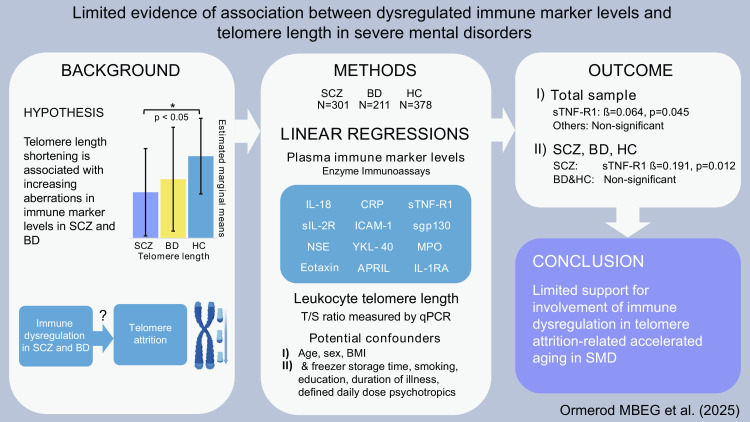

Significant outcomes

- Accelerated ageing indexed by TL attrition is suggested in SCZ.
- A positive association between TL and levels of sTNF-R1 in SCZ was observed.
- No associations between TL and most immune markers in total sample, SCZ, BD or HC.

Limitations

- Immune marker selection might fail in capturing immune signalling associated with TL attrition.
- A relatively young participant sample might reduce the ability to detect associations with TL.
- Potential residual confounding factors cannot be ruled out despite extensive adjustments.

## Introduction

Schizophrenia spectrum disorders (SCZ) and bipolar disorders (BD) are severe mental disorders (SMDs) with overlapping clinical characteristics and etiopathogenic factors (Mccutcheon *et al*., 2020; Mcintyre *et al*., 2020). SMDs are associated with a shortened life expectancy of about 15 years (Hjorthoj *et al*., 2017; Chan *et al*., 2022), mainly caused by suicide and somatic comorbidities such as cardiovascular disease (Correll *et al*., 2022; Biazus *et al*., 2023). While lifestyle factors and cardiometabolic effects of psychotropic agents are established risk factors of the excessive somatic comorbidity and mortality (Dieset *et al*., 2016), accelerated ageing is a less studied candidate (Kirkpatrick *et al*., 2008; Lima *et al*., 2015; Teeuw *et al*., 2021).

Telomere attrition of chromosomes during cell divisions entails cell senescence at a critical telomere length (TL) (Rode *et al*., 2015). Senescent cells accumulate in proliferative tissue during ageing (Rossiello *et al*., 2022), and TL shortening in leucocytes correlates positively with age and mortality in the general population (Rode *et al*., 2015). TL shortening is used as an indicator of accelerated ageing and is demonstrated in the majority of studies of SMDs (Simon *et al*., 2006; Elvsashagen *et al*., 2011; Rizzo *et al*., 2013; Lima *et al*., 2015; Lindqvist *et al*., 2015; Pawelczyk *et al*., 2015; Barbe-Tuana *et al*., 2016; Darrow *et al*., 2016; Rao *et al*., 2016; Maurya *et al*., 2017; Wolkowitz *et al*., 2017; Russo *et al*., 2018; Vakonaki *et al*., 2018; Aas *et al*., 2019; Birkenaes *et al*., 2021). However, a few studies report longer (Nieratschker *et al*., 2013; Maurya *et al*., 2018) or similar TL in SCZ relative to controls (Lindqvist *et al*., 2015; Polho *et al*., 2015; Cevik *et al*., 2019; Schurhoff *et al*., 2021). Longer TL is reported in patients with BD using lithium compared to non-lithium users (Martinsson *et al*., 2013; Squassina *et al*., 2016; Coutts *et al*., 2019; Pisanu *et al*., 2020). While the indicated increased telomere attrition in SCZ and BD supports accelerated ageing, the biological processes underlying the TL abnormalities are unclear.

Immune abnormalities and inflammation may be involved in the pathophysiology of SCZ and BD as evidenced by molecular genetics, central and peripheral biological associations, and epidemiological data (Miller and Goldsmith, 2017; Kroken *et al*., 2018; Chen *et al*., 2024). Associations with the immune-related major histocompatibility complex (MHC) locus and with immune loci outside of the MHC region are demonstrated in genome-wide association studies of both disorders (Andreassen *et al*., 2013; Pouget, 2018; Mullins *et al*., 2021; Trubetskoy *et al*., 2022). SCZ and BD share genetics with immune-mediated diseases such as cardiovascular disease, multiple sclerosis and inflammatory bowel disease (Andreassen *et al*., 2013; Andreassen *et al*., 2015; Kember *et al*., 2018; Pouget *et al*., 2019; Rodevand *et al*., 2021). Brain imaging and markers in cerebrospinal fluid and post-mortem brain tissue indicate low-grade neuroinflammation in SMDs (Bechter *et al*., 2010; Trepanier *et al*., 2016; Marques *et al*., 2019; Benedetti *et al*., 2020; Giridharan *et al*., 2020), and low-grade systemic inflammation is evidenced by a range of blood immune marker studies (Goldsmith *et al*., 2016; Muneer, 2016; Frydecka *et al*., 2018; Khoury and Nasrallah, 2018; Kroken *et al*., 2018; Benedetti *et al*., 2020). Systemic immune pathways and markers typically associated with SMDs include tumour necrosis factor (TNF), interleukin (IL)-1, -2, -6 and -18 signalling (Goldsmith *et al*., 2016; Kroken *et al*., 2018), adhesion molecules (Muller, 2019), C-reactive protein (CRP) (Lestra *et al*., 2022) and chemokines (Misiak *et al*., 2020; Ermakov *et al*., 2023). Lastly, large epidemiological studies of SMDs demonstrate co-occurrence with autoimmune disorders and severe infections as risk factors (Benros *et al*., 2011; Bergink *et al*., 2014; Najjar *et al*., 2018; Cullen *et al*., 2019; Köhler-Forsberg *et al*., 2019).

Chronic low-grade inflammation has been reported to induce telomere attrition and accelerated senescence by enhancing cell turnover, as well as to induce telomere and DNA damage by increasing reactive oxygen species load (Jurk *et al*., 2014; Barnes *et al*., 2019). Accelerated telomere attrition is associated with various immune-related somatic conditions, including infections, cardiometabolic and autoimmune disorders (Zhang *et al*., 2016; Squassina *et al*., 2019). A bidirectional relationship is also suggested, with telomere attrition-mediated immune dysregulation and inflammation involving the production of pro-inflammatory cytokines by senescent cells (Zhang *et al*., 2016; Lustig *et al*., 2017; Rossiello *et al*., 2022). In SMDs, some studies suggest that systemic immune abnormalities and inflammation are involved in TL abnormalities. Negative associations between TL and levels of the chemokine eotaxin and high-sensitivity CRP have been reported in SCZ (Czepielewski *et al*., 2018) and a mixed sample of SCZ, BD, major depressive disorder and non-psychiatric controls (Squassina *et al*., 2020), respectively. However, few studies have been conducted and the sample sizes are small (Lindqvist *et al*., 2015; Squassina *et al*., 2019).

To identify potential underlying mechanisms of accelerated telomere attrition in SCZ and BD, we aimed to investigate associations between levels of peripheral immune markers with established links to these disorders and leucocyte TL. We hypothesised that a shortening of telomeres was associated with increasing aberrations in immune marker levels in SCZ and BD, in line with the concept of immune dysregulation involvement in the causal mechanism of accelerated telomere attrition. Twelve immune markers reflecting well-studied immune pathways in SCZ and BD were analysed, including soluble tumour necrosis factor receptor 1A (sTNF-R1) (Morch *et al*., 2017), interleukin-1 receptor antagonist (IL-1Ra) (Hope *et al*., 2015; Morch *et al*., 2017; Werner *et al*., 2022a), IL-18 (Hjell *et al*., 2022), soluble interleukin-2 receptor (sIL-2R) (Werner *et al*., 2022a), soluble glycoprotein 130 (sgp130) (Aas *et al*., 2017), intercellular adhesion molecule -1 (ICAM-1) (Werner *et al*., 2022b), a proliferation-inducing ligand (APRIL) (Engh *et al*., 2021), chitinase-3-like protein 1 (YKL-40) (Dieset *et al*., 2019), myeloperoxidase (MPO) (Reponen *et al*., 2020), neuron specific enolase (NSE) (Andreou *et al*., 2021), CRP (Dieset *et al*., 2015) and eotaxin (Teixeira *et al*., 2018). Healthy controls (HC) were included as a comparison group. The analyses were adjusted for age, sex (Wolkowitz *et al*., 2017) and body mass index (BMI) (Gielen *et al*., 2018), followed by sensitivity analyses of suggested associations with further adjustments.

## Methods

### Study setting

The current sample is based on inclusion of patients and HC to the Thematically Organized Psychosis (TOP) study at the Norwegian Center for Mental Disorder Research (NORMENT). Patients meeting the Diagnostic and Statistical Manual of Mental Disorders (DSM)-IV (First, 2013) criteria for SCZ or BD are recruited on an ongoing basis from the major hospitals in Oslo, and HC are recruited based on random selection from the same catchment area. The TOP study has invested major efforts to achieve a comprehensive inclusion of patient participants from in- and outpatient clinics in a medium-sized transcultural capital Oslo with a wide range of sociodemographic differences and equal access to public health care services. Individuals between 18 to 65 years of age who have sufficient Scandinavian language skills to complete the study protocol are asked to participate. Exclusion criteria are severe somatic illness potentially interfering with brain functioning, including neurological disorders, history of severe head trauma, and IQ<70. Additionally, HC with current substance abuse or dependency, or with close relatives with SMDs, are excluded.

### Sample

Participants from the TOP study with measurements of immune marker levels and TL (*N* = 890), comprising *N* = 301 patients with SCZ (schizophrenia, *N* = 162; schizophreniform disorder, *N* = 24; schizoaffective disorder, *N* = 49; psychotic disorder not otherwise specified, *N* = 66), *N* = 211 patients with BD (bipolar I disorder, *N* = 137; bipolar II disorder, *N* = 58; bipolar disorder not otherwise specified, *N* = 16) and *N* = 378 HC were included. Sixteen individuals were excluded from the analyses as the blood sampling did not coincide for immune markers and TL, and 54 participants were excluded due to CRP levels above 10.0 mg/L, to prevent impact from acute infections on immune marker levels (Fathian *et al*., 2022).

### Clinical assessments

Trained clinical psychologists and medical doctors performed clinical interviews, obtaining sociodemographic, psychiatric and somatic information. Diagnostic interviews were conducted using the Structured Clinical Interview for DSM-IV Axis I Disorders (SCID-1) (Spitzer *et al*., 1992). Inter-rater reliability of the diagnostics has previously been estimated to an overall kappa score between 0.92 and 0.99 (Høegh *et al*., 2022). Present symptom severity was evaluated with the Positive and Negative Syndrome Scale (PANSS) (Kay *et al*., 1987). Within two weeks of assessing symptom severity, a somatic examination including height and weight for BMI, and routine blood tests was performed. Information about prescribed medication was collected from interviews and medical records, and defined daily dosages (DDD) (World Health Organization Collaborating Centre for Drug Statistics Methodology, 2024) of antipsychotic agent (AP) use, antidepressant agent (AD) use and mood stabilising agent (MS; antiepileptics and lithium) use were calculated (Table 1). Details of anti-inflammatory, cardiometabolic and other somatic agent use are given in Supplementary Table 1.

**Table 1.** Sample descriptives

	SCZ (N = 301)	BD (N = 211)	HC (N = 378)	*p*-value^a^	Post hoc
*Clinical characteristics*					
Sex (males), N (%)	178 (59.1)	90 (42.7)	218 (57.7)	**<0.001**	BD < HC, SCZ
Age (years), median (IQR)	27.0 (12.0)	28.0 (16)	31.0 (13.0)	**<0.001**	SCZ < HC
BMI (kg/m2), median (IQR)	25.55 (7)	25.42 (6.0)	23.9 (4.00)	**<0.001**	HC < SCZ, BD
Smoking (yes), N (%)	143 (52.2)	83 (30.3)	48 (17.5)	**<0.001**	HC < SCZ, BD
Education (years), median (IQR)	13.0 (4.0)	14 (5.0)	–	**<0.001**	SCZ < BD
Duration of illness (years), median (IQR)	4.0 (8.0)	8.0 (11.0)	–	**<0.001**	SCZ < BD
PANSS total score, median (IQR)	63.0 (21.0)	44.0 (12)	–	**<0.001**	BD < SCZ
Freezer storage time (years), median (IQR)	8.0 (6.0)	7.0 (6.0)	5.0 (5.0)	**<0.001**	HC < SCZ, BD
Telomere length ratio, median (IQR)	1.25(0.76)	1.31 (0.73)	1.37 (0.60)	**0.033**	SCZ < HC
DDD AP agent use, median (IQR)	1.00 (1.17)	0.13 (0.88)	–	**<0.001**	BD < SCZ
DDD AD agent use, median (IQR)	0 (0.61)	0 (1.00)	–	0.29	–
DDD MS agent use, median (IQR)	0 (0.0)	0 (1.00)	–	**<0.001**	SCZ < BD
*Immune markers*					
sTNF-R1 (ng/mL), median (IQR)	1.75 (0.74)	1.70 (0.59)	1.75 (0.88)	0.88	–
IL-1RA (pg/mL), median (IQR)	214 (269)	178 (261)	243 (253)	**0.045**	BD < HC
sIL-2R (ng/mL), median (IQR)	0.28 (0.18)	0.25 (0.19)	0.25 (0.10)	**0.025**	HC < SCZ
sgp130 (ng/mL), median (IQR)	208 (62.5)	202 (45.2)	230 (64.2)	**<0.001**	SCZ, BD < HC
ICAM-1 (ng/mL), median (IQR)	281 (116)	280 (105)	255 (94.3)	**<0.001**	HC < SCZ
IL-18 (pg/mL), median (IQR)	1114 (1839)	650 (1705)	1254 (1411)	**0.002**	BD < SCZ, HC
APRIL (pg/mL), median (IQR)	273 (267)	242 (194)	369 (261)	**<0.001**	SCZ, BD < HC
YKL-40 (ng/mL), median (IQR)	33.3 (20.8)	35.8 (24.2)	32.0 (20.0)	**0.014**	HC < BD
MPO (ng/mL), median (IQR)	172 (249)	188 (335)	227 (308)	**0.039**	SCZ < HC
NSE (ng/mL), median (IQR)	2.35 (3.80)	2.42 (4.39)	5.17 (7.09)	**<0.001**	SCZ, BD < HC
CRP (mg/L), median (IQR)	1.15 (1.97)	1.03 (2.30)	0.90 (1.05)	**<0.001**	HC < SCZ, BD
Eotaxin (pg/mL), median (IQR)	2.13 (0.29)	2.13 (0.23)	2.16 (0.22)	**0.005**	SCZ < HC

a Chi square test for categorical variables, Kruskal–Wallis and Mann–Whitney *U*-Test for variables represented by median (IQR).

Missing data, N (%): BMI 42 (4.7), smoking 192 (21.6), education 4 (0.5), duration of illness 10 (1.1), PANSS 9 (1.0), freezer storage time 21 (2.4), ICAM-1 and APRIL 18 (2.0), IL-18 57 (6.8), NSE 17 (2.0), eotaxin 19 (2.1).

### Immune markers

Blood samples were drawn on EDTA vials and plasma was isolated within the next working day and stored at -80 degrees Celsius for later analyses. Typical time of blood sampling were 09:15 (median, min 07:30, max 15:15) for patients and 10:50 (median, min 08:00, max 18:10) for HC (Morch *et al*., 2016; Hjell *et al*., 2023). Samples were analysed in duplicate with enzyme immunoassays at the Research Institute of Internal Medicine, Oslo University Hospital using antibodies from R&D systems (Minneapolis, MN, USA) in a 384 format by combining use of a Selma pipetting robot and a Biotek dispenser/washer. An ELISA plate reader (BIO-RAD, Hercules, CA, USA) was used to read absorbance at 450 nm with wavelength correction at 540 nm. Intra- and inter-assay coefficients of variation were<10 % in all EIAs. All plasma samples went through one freeze/thaw cycle prior to analysis of immune markers. While the chosen markers are in general circulating at measurable levels (e.g. cell adhesion molecules and soluble receptors), some proteins had levels below the limit of detection (LLOD) (Armbruster and Pry, 2008); these were set to the LLOD. This includes 2 samples for NSE (set to 100 pg/mL), 11 samples for IL-1RA (set to 25 pg/mL), 5 samples for IL-18 (set to 125 pg/mL) and 3 samples for APRIL (set to 50 pg/mL).

The immune markers were chosen based on documented associations with SMDs (Potvin *et al*., 2008; Drexhage *et al*., 2010; Goldsmith *et al*., 2016) and constitute sTNF-R1, IL-1RA, sIL-2R, sgp130, IL-18, ICAM-1, APRIL, YKL-40, MPO, NSE, CRP and eotaxin (George-Chandy *et al*., 2008; Palladino *et al*., 2012; Hope *et al*., 2013; Aas *et al*., 2017; Dinarello, 2018; Teixeira *et al*., 2018; Muller, 2019; Andreou *et al*., 2021; Lestra *et al*., 2022). IL-1RA (Palomo *et al*., 2015; Goldsmith *et al*., 2016), IL-18 (Ihim *et al*., 2022), sIL-2R (Goldsmith *et al*., 2016) and sgp130 (subunit of IL-6 receptor) (Jones and Jenkins, 2018) are components of cytokine inflammatory pathways; ICAM-1 is an adhesion molecule mediating inflammation and leucocyte transmigration, reflecting among other things blood-brain barrier (BBB) integrity (Muller, 2019); the cytokine APRIL is involved in B- and T-cell regulation (Engh *et al*., 2021); YKL-40 is an inflammatory marker associated with first-episode psychosis (Orhan *et al*., 2018); the innate immunity enzyme MPO is particularly produced by neutrophils and involved in oxidative stress (Ndrepepa, 2019); NSE is an enzyme suggested to reflect neuronal stress and neural maturation (Haque *et al*., 2018; Andreou *et al*., 2021); the commonly used acute-phase protein CRP reflects unspecific inflammation and is stimulated by TNF, IL-6 and IL-1β (Sproston and Ashworth, 2018); and lastly the chemokine eotaxin reflects inflammation by eosinophil recruitment and has recently been proposed as an ageing biomarker in SMDs (Teixeira *et al*., 2018). sTNF-R1, IL-1RA, sIL-2R, YKL-40, MPO and sgp130 were analysed in a subsample in 2013 (60.1 % of the total sample) (e.g. Morch *et al*., 2016), while the other immune markers were analysed in the total sample in 2018 (e.g. Ormerod *et al*., 2022), see Table 1 legend for further details.

### Telomere length (TL)

TL was defined by the ratio telomere template/amount of single-copy gene template (T/S ratio), hence, TL relative to standard reference DNA (Kam *et al*., 2021). Smaller T/S ratio indicates shorter average TL. TL was measured in peripheral leukocytes in blood drawn on Tempus Blood RNA-tubes (Life Technologies Corporation) as per standard practice (Akkouh *et al*., 2018). The samples were stored at -80 degrees Celsius before analyses with a modified quantitative real-time polymerase chain reaction (qPCR) at the Newcastle University BioScreening Core Facility-CAV. The analysis procedure is previously described (Aas *et al*., 2019). The qPCR estimated the large quantity of a single-copy gene (36B4) versus telomeric template on 10 ng of DNA with 0.25 µL of ROX reference dye (Sigma-Aldrich, Gillingham, UK) and 5 µL SYBR®Green JumpStart Taq Ready Mix. Primers for the telomeric reaction were 300 nM TelA (5′-CGG TTT GTT TGG GTT TGG GTT TGG GTT TGG GTT TGG GTT-3′) and 900 nM TelB (5′-GGC TTG CCT TAC CCT TAC CCT TAC CCT TAC CCT TAC CCT-3′). Primers for 36B4 were 200 nM 36B4F (5′-CAG CAA GTG GGA AGG TGT AAT CC 3′) and 400 nM 36B4R (5′-CCC ATT CTA TCA ACG GGT ACA A-3′). PCRs were performed on an Applied Biosystems 7900HT Fast Real Time qPCR system with 384-well plate capacity, and the samples were assessed in triplicate. Plate-to-plate variation was corrected for by running three internal control DNA samples of known TL (2 kb, 3.9 kb, 10.4 kb) within each plate. To ensure TL measurement accuracy, TL measurement samples in the bottom or top 5 % were reassessed, in addition to samples with initial invalid data. The inter- and intra-assay coefficient of variation was 6.08 % and 6.07 %, respectively (Aas *et al*., 2019). Analysis of the TL data from the TOP study has previously been published (Aas *et al*., 2019; Birkenaes *et al*., 2021; Mlakar *et al*., 2023). One case qualified as an outlier in terms of TL and was excluded from the statistical analyses.

### Statistical analysis

Statistical analyses were performed with the Statistical Package for the Social Sciences (SPSS) for Windows version 29 (SPSS Inc., Chicago, IL, USA). Sample characteristics (Table 1) were analysed with chi-square tests for categorical variables, and Kruskal–Wallis test and Mann–Whitney *U*-test for continuous variables. Normality of TL and immune marker data was evaluated by use of histograms, Q-Q-plots, and Kolmogorov-Smirnov statistics. Linear regression was used to analyse associations between immune markers (independent variable) and TL (dependent variable). To reduce the total number of analyses performed and the risk of spurious findings, we first screened the total sample for suggested statistical effects (*p*<0.1) of immune markers on TL; only immune markers identified in this first step were included in the main analyses. In the main analyses, immune markers were, according to the hypotheses, analysed separately with TL in each group, to test whether immune marker levels were associated with TL in SCZ and BD. Findings in the HC group, as being less prone to biasing effects due to recruitment based on random selection, served to validate the findings in patients. We corrected for multiple testing in the main analyses by applying a moderate significance threshold of *p*<0.017 (0.05/3, cf. analyses in three separate groups) to avoid a too conservative threshold, which could lead to a high risk of rejecting relevant associations in this less investigated field. Statistical adjustments in the total sample and main analyses included the variables age, sex and BMI. To scrutinise associations in patients, we further conducted sensitivity analyses of the associations tested in the main analyses by performing extensive adjustments, additionally including the variables smoking (yes/no), education (years), duration of illness (years), PANSS total score, freezer storage time (years) and DDDs of AP, AD and MS. We also performed explorative analyses of sex-specific immune marker and TL associations.

### Ethics

Participation in the TOP study is voluntary and based on written informed consent. The TOP study is approved by the Regional Committee for Medical and Health Research Ethics (2009/2485).

## Results

### Sample characteristics

Unadjusted analyses of sample characteristics showed that the BD group had significantly fewer male participants than the HC and SCZ groups (both *p*<0.001), and that SCZ participants on average were younger than HC (*p*<0.001). HC had lower BMI compared to SCZ and BD (both *p*<0.001). Plasma levels of the immune markers differed between SCZ and/or BD and healthy controls (*p*<0.05), except for sTNF-R1. The T/S ratio was significantly lower in SCZ than in HC [*p* = 0.010, (see Supplementary Figure 1 for the covariate adjusted difference)]. The T/S ratio was not significantly different in BD vs. HC or in BD vs. SCZ, and these characteristics did not change when excluding participants with BD using lithium. Table 1 shows sample characteristics in more detail, including variables used in the sensitivity analyses. Data on differences between cases and controls of immune marker levels and TL from the total TOP-sample is previously published elsewhere (e.g. Aas *et al*., 2019; Ormerod *et al*., 2022; Werner *et al*., 2022b).

### Associations of TL with immune marker levels

**Figure 1.**
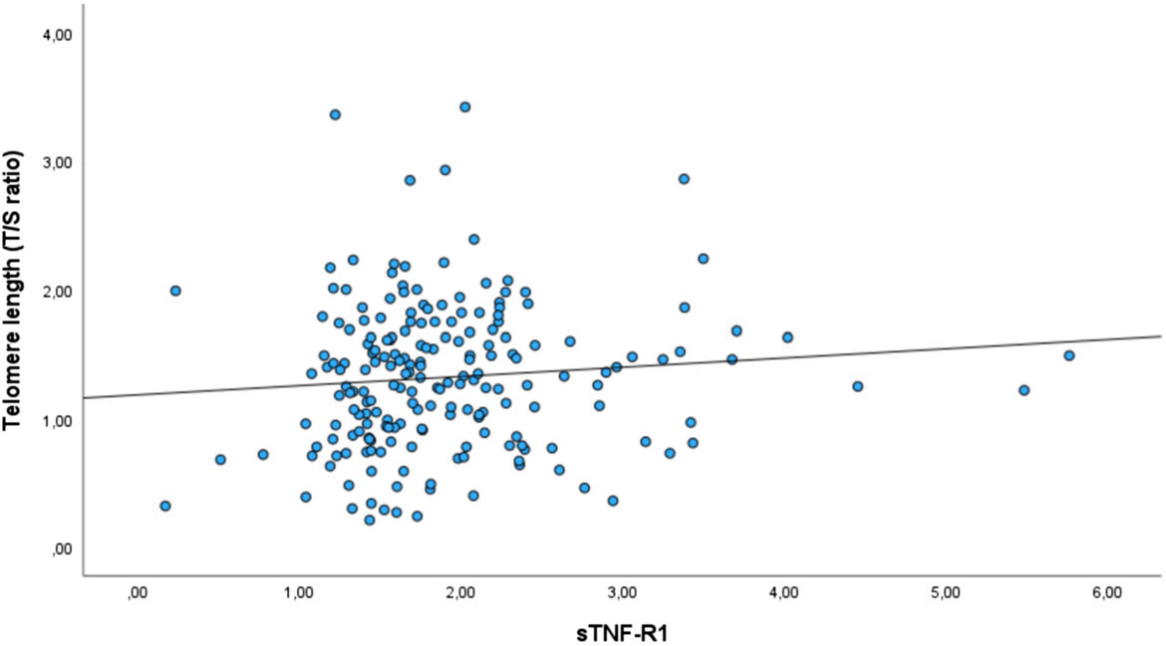
Association between levels of sTNF-R1 (ng/mL) and telomere length in SCZ (raw data). A smaller T/S ratio equals shorter telomere length. Abbreviations: schizophrenia spectrum disorders (SCZ), soluble tumor necrosis factor receptor 1 (sTNF-R1), telomere template/amount of single-copy gene template (T/S ratio).

Two immune markers were identified for further analysis from the total sample analyses [sTNF-R1 (*β* = 0.064, *p* = 0.045) and IL-1RA (*β* < -0.001, *p* = 0.075), Table 2]. The associations between these immune markers and TL were non-significant in analyses of the SCZ, BD and HC groups separately [Table 3 (see Supplementary Table 2 for analyses in the combined patient group)]. In the sensitivity analyses performed with extensive adjustments, a significant association between sTNF-R1 and T/S ratio in SCZ (β = 0.191, *p* = 0.012) was found (Table 4, Fig. 1).

**Table 2.** Association analyses of immune markers and telomere length^a^ in total sample

	Model: Adjustments for age, sex, BMI	
	*ß*	*p-value*
sTNF-R1	0.064	0.045
IL-1RA	<−0.001	0.075
sIL-2R	0.002	0.83
sgp130	0	0.56
ICAM-1	<−0.001	0.80
IL-18	<0.001	0.94
APRIL	<−0.001	0.57
YKL-40	0	0.80
MPO	<−0.001	0.84
NSE	0.005	0.19
CRP	0.007	0.56
Eotaxin	<0.001	0.61

a Telomere length is defined by the ratio telomere template/amount of single-copy gene template (T/S ratio). Body mass index (BMI).

**Table 3.** Association analyses of immune markers and telomere length^a^ in SCZ vs. BD vs. HC

	Model: IM and adjustment for age, sex and BMI			Model^b^: IM × sex interaction and adjustment for age, sex and BMI			Model^c^: IM and adjustment for age and BMI		
	SCZ	BD	HC	SCZ	BD	HC	SCZ	BD	HC
	*ß (p)*	*ß (p)*	*ß (p)*	*ß (p)*	*ß (p)*	*ß (p)*	*ß (p)*	*ß (p)*	*ß (p)*
sTNF-R1	0.095 (0.09)	0.095 (0.22)	−0.009 (0.84)	−0.001 (0.99)	0.169 (0.28)	−0.216 (**0.01**)	–	–	0.096 (0.080)/ −0.123 (0.056)
IL-1RA	<−0.001 (0.07)	<−0.001 (0.29)	<0.001 (0.66)	<−0.001 (0.72)	<0.001 (0.42)	<−0.001 (0.85)	–	–	–

a Telomere length is defined by the ratio telomere template/amount of single-copy gene template (T/S ratio).

b Results shown for the interaction term.

c Results shown as males/females.

Bipolar disorder (BD), Body mass index (BMI), Healthy controls (HC), Immune marker (IM), Schizophrenia spectrum disorders (SCZ).

**Table 4.** Association analyses of immune markers and telomere length, sensitivity analyses in SCZ and BD

	Model: Extended number of potential confounders^a^			
	SCZ		BD	
	*ß*	*p-value*	*ß*	*p-value*
sTNF-R1	0.191	**0.012**	0.081	0.411
IL-1RA	< -0.001	0.077	< -0.001	0.312

a Model: adjustment for age, sex, BMI, smoking, education, duration of illness, PANSS total score, freezer storage time, DDD AP, DDD AD, DDD MS.

Antidepressant agents (AD), Antipsychotic agents (AP), Bipolar disorder (BD), Body mass index (BMI), Defined daily dosage (DDD), Mood stabilising agents (MS), Positive and Negative Syndrome Scale (PANSS), Schizophrenia spectrum disorder (SCZ).

There were no interaction effects between immune markers and sex in SCZ or BD. In HC there was a significant interaction effect between sex and levels of sTNF-R1 (*p* = 0.01); however, sex-stratified analyses revealed no significant associations (Table 3).

## Discussion

In the current study we found a positive significant association between sTNF-R1 and TL in SCZ, and no significant associations between TL and the other immune markers. These results provide limited support for the hypothesis that immune abnormalities and inflammation are involved in accelerated ageing in SCZ and BD, as measured with TL and peripheral immune markers.

sTNF-R1 is part of the TNF-signaling system as a circulating soluble subunit of the TNF-receptor, acting anti-inflammatory by binding TNF-α, thus antagonising activation of the TNF-pathway (Paccalet *et al*., 2021). sTNF-R1 levels increase as a regulatory response to inflammation, and is released from most cells, including microglia, astrocytes and neurones (Kroken *et al*., 2018; Salomon, 2021). The positive association between levels of sTNF-R1 and TL in SCZ in the sensitivity analyses should be interpreted with caution, particularly as sTNF-R1 levels were not significantly dysregulated in SCZ. However, due to the anti-inflammatory action, one might speculate of a compensatory protective effect on TL. In comparison, a positive association was suggested between plasma levels of TNF-α and advanced brain ageing in SCZ by Klaus *et al*. (2022). The study also reported the absence of any noteworthy links between CRP, IL-6, ICAM-1, eotaxin and advanced brain ageing (Klaus *et al*., 2022), in line with the current results. Thus, based on the association of sTNF-R1 and TL, we cannot exclude the possibility of the involvement of disorder-linked immune abnormalities, particularly TNF-α signalling, in mechanisms underlying regulation of the accelerating ageing as indexed by telomere attrition in SCZ.

However, overall, the current findings provide little support for immune dysregulation, as reflected by circulating levels of a range of inflammatory markers, playing a significant role in accelerated ageing as indexed by telomere attrition, in SCZ and BD. Specifically, 1) testing immune markers and inflammatory pathways robustly linked to SMDs, obtaining 2) similar findings across separate patient groups sharing etiopathogenic mechanisms (Tamminga *et al*., 2013), which are 3) paralleled by similar negative findings in the randomly recruited HC group, indicate valid findings. Moreover, to the best of our knowledge, this is the largest single study examining potential immune involvement in telomere attrition in SMDs including both SCZ and BD, and the results are based on well-adjusted analyses.

In contrast, the previous evidence in SMDs suggests involvement of immune dysregulation and inflammation in accelerated ageing (Solana *et al*., 2018; Squassina *et al*., 2019; Fries *et al*., 2020). Four studies report links between immune markers and accelerated ageing in BD; however, sample sizes are small and with no specific testing related to lithium use (Rizzo *et al*., 2013; Panizzutti *et al*., 2015; Vasconcelos-Moreno *et al*., 2017; Mohite *et al*., 2020). Also, these studies contrast the current study by the sample of Rizzo *et al*. (2013) being restricted to women, Panizzutti *et al*. (2015) analysing eotaxin but not TL as a potential ageing biomarker, Vasconcelos-Moreno *et al*. (2017) indicating an association between TL and levels of pro-inflammatory markers without conducting specific association analyses, and the study by Mohite *et al*. (2020) lacking adjustments for variables such as age, sex and psychotropic agents. In SCZ, a study of about one eighth the sample size of ours suggested eotaxin to be negatively correlated with TL, with additional associations to reduced grey matter volume (Czepielewski *et al*., 2018). Moreover, a proteomics and metabolomics study in SCZ reported dysregulation of inflammatory components associated with somatic ageing diseases; however, TL was not analysed (Campeau *et al*., 2022). Interestingly, a negative association was reported between TL and high-sensitivity CRP in a study of SCZ, BD, major depressive disorder (MDD) and non-psychiatric controls; however, the study did not report diagnosis-specific associations (Squassina *et al*., 2020). While increased levels of eotaxin has been associated with SMDs and accelerated ageing (Czepielewski *et al*., 2018; Teixeira *et al*., 2018), we found decreased eotaxin levels in SCZ, but no significant association with TL, thus, questioning the role of eotaxin as a marker of accelerated ageing in SMDs. Importantly, our conclusion for the immune marker and TL associations were unchanged also after combining the SCZ and BD groups in one SMDs group. By comparison, significant negative correlations between IL-6, TNF-α and CRP levels and TL have been reported in a large sample of individuals with MDD (Révész *et al*., 2014). These results were in line with another, smaller study in which TL shortening associated with cumulative lifetime exposure to MDD, influenced by chronic inflammation, was suggested (Wolkowitz *et al*., 2011). Similarly, associations between high IL-6 and TNF-α levels but not CRP and cumulative effect of chronic inflammation, and TL shortening, have been reported in HC (O’donovan *et al*., 2011), particularly in individuals exposed to childhood trauma (Kiecolt-Glaser *et al*., 2011). The MDD and HC studies included somewhat older individuals than the current study, and we can only speculate that the younger age might have prevented us from detecting additional significant associations.

We have previously reported differences in TL between SMDs and HC in a sample partly overlapping with the current sample (Aas *et al*., 2019). In BD, other studies have reported both shorter (Simon *et al*., 2006; Elvsashagen *et al*., 2011; Rizzo *et al*., 2013; Lima *et al*., 2015; Barbe-Tuana *et al*., 2016; Darrow *et al*., 2016; Vasconcelos-Moreno *et al*., 2017; Huang *et al*., 2018), longer (Squassina *et al*., 2020) and no differences in TL (Mamdani *et al*., 2015; Fries *et al*., 2017; Palmos *et al*., 2018; Mutz and Lewis, 2023). However, lithium use might impact TL (Coutts *et al*., 2019; Pisanu *et al*., 2020). In the current BD sample, sensitivity analysis by excluding patients using lithium, indicated the robustness of the finding of no TL alteration. Still, based on the numerical intermediate position of TL in BD (Table 1, Supplementary Figure 1), one might speculate about a minor TL shortening that would require larger sample sizes for detection. Although supported by a recent meta-analysis (Ayora *et al*., 2022), the T/S ratio in SCZ was only borderline significant in our study, and the impact on longevity in this population is not evident. However, high variability in TL across tissues has been reported (Dlouha *et al*., 2014) and even across brain regions (Mamdani *et al*., 2015). Moreover, brain-predicted age based on MRI estimates suggests advanced structural brain age in SCZ (Constantinides *et al*., 2023) and BD (Ballester *et al*., 2022). Thus, future studies should evaluate associations between immune abnormalities and inflammation as assessed by circulating markers and accelerated brain-aging based on MRI assessment. In addition, other mechanisms influencing TL including stress, lifestyle and behavioural factors such as physical inactivity and sleep problems (Lin *et al*., 2012; Qiao *et al*., 2020), should be addressed.

Strengths of the present study is the sizable and well-characterized sample enabling comprehensive statistical adjustments, and the investigation of immune pathways with established associations to SMDs and markers of neuroinflammation, BBB integrity and neurodevelopment (Goldsmith *et al*., 2016). Some of the current immune markers are less investigated in SMDs, such as sgp130 and sTNF-R1, but reflect much investigated pathways (Solmi *et al*., 2021; Halstead *et al*., 2023). We cannot rule out that analysis of other, more commonly investigated markers, such as IL-6 and TNF-α, might have provided different results. Moreover, we cannot exclude that the selection failed to detect immune signalling associated with telomere attrition in SMD, as it is unlikely that we captured the full scope of immune pathways influencing TL. This might explain the discrepancy to findings in a few other studies. Given that immune dysregulation is present in a subset of individuals with SMDs, associations might have been identified in analyses stratified by inflammatory state. Moreover, the complex and dynamic interplay among the factors involved, such as the proposed bidirectional relationship between inflammation and telomere attrition and senescence, complicates the interpretation and might impede our ability to detect actual effects. Both TL and immune markers were measured peripherally, and the findings might not reflect processes in specific tissues of importance in SMDs, including the central nervous system. Furthermore, all plasma samples went through one freeze/thaw-cycle prior to the analysis of immune markers, which may have affected the measured levels. However, all samples went through the same cycle, which reduces the probability of a significant impact on the investigated associations. Although the sample was adjusted for a range of potential confounders of the TL and immune marker associations, we cannot exclude the possibility of residual confounding that might have impacted the results. However, similar findings in the patient and HC groups support the main findings. The cross-sectional design inhibits causal inferences. Lastly, we cannot rule out false negative results, for example caused by the relatively young participants in the sample, which might have reduced the ability to detect actual associations with TL compared to a sample of older participants.

In the present study we found few significant associations between levels of peripheral immune markers and TL in SMDs, despite the large sample size and extensive adjustment for potential confounders. Thus, the results provide limited support for immune dysregulation and inflammation contributing to accelerated ageing as indexed by telomere attrition in SCZ or BD.

## Supporting information

Ormerod et al. supplementary material Ormerod et al. supplementary material

## Supplementary material

The supplementary material for this article can be found at https://doi.org/10.1017/neu.2024.62.
