# Supplementary material for: Limited evidence of association between dysregulated immune marker levels and telomere length in severe mental disorders
Source: Acta Neuropsychiatr. 2025 Jan 23;37:e4. doi: 10.1017/neu.2024.62 (PMC13130312; doi:10.1017/neu.2024.62)
Supplement: Ormerod et al. supplementary material [file S0924270824000620sup001.docx]

**Ormerod MBEG et al.,** **Limited evidence of association between dysregulated immune marker levels and telomere length in severe mental disorders**

Supplementary material

Content

- **Supplementary Table 1** Somatic medication use by patients
- **Supplementary Table 2** Association analyses of immune markers and telomere length in the combined SCZ + BD group
- **Supplementary Figure 1** Difference in telomere length by T/S ratio among SCZ, BD and HC

| **Supplementary Table 1** Somatic medication use by patients | | |
| --- | --- | --- |
| **Somatic medications** | SCZ  (N = 301) | BD  (N = 211) |
| Anti-inflammatory/immunomodulatory, N (%) | 17 (2.8) | 8 (2.3) |
| Antidiabetics, N (%) | 10 (1.7) | 4 (1.2) |
| Cardiovascular/lipid modifying, N (%) | 19 (3.2) | 15 (4.3) |
| Gastrointestinal drugs, N (%) | 21 (3.5) | 8 (2.3) |
| Other, N (%) | 86 (14.3) | 74 (21.4) |
| Abbreviations: Bipolar disorder (BD), Schizophrenia spectrum disorders (SCZ). | | |

| **Supplementary Table 2** Association analyses of immune markers and telomere length in the combined SCZ + BD group | | | |  |
| --- | --- | --- | --- | --- |
|  | **Model: IM and adjustment for** **age, sex and BMI** | | | |
|  | SCZ + BD | |  |  |
|  | *ß_(IM)_* | *p-value* |  |  |
| sTNF-R1 | 0.097 | 0.035 |  |  |
| IL-1RA | <-0.001 | 0.025 |  |  |
| sIL-2R | -0.002 | 0.86 |  |  |
| sgp130 | 0.001 | 0.17 |  |  |
| ICAM-1 | <0.001 | 0.38 |  |  |
| IL-18 | <0.001 | 0.92 |  |  |
| APRIL | <-0.001 | 0.66 |  |  |
| YKL-40 | <0.001 | 0.93 |  |  |
| MPO | <0.001 | 0.90 |  |  |
| NSE | 0.007 | 0.32 |  |  |
| CRP | 0.009 | 0.58 |  |  |
| Eotaxin | <-0.001 | 0.83 |  |  |
| Abbreviations: Bipolar disorder (BD), Body mass index (BMI), Healthy controls (HC), Immune marker (IM), Schizophrenia spectrum disorders (SCZ). | | | |  |


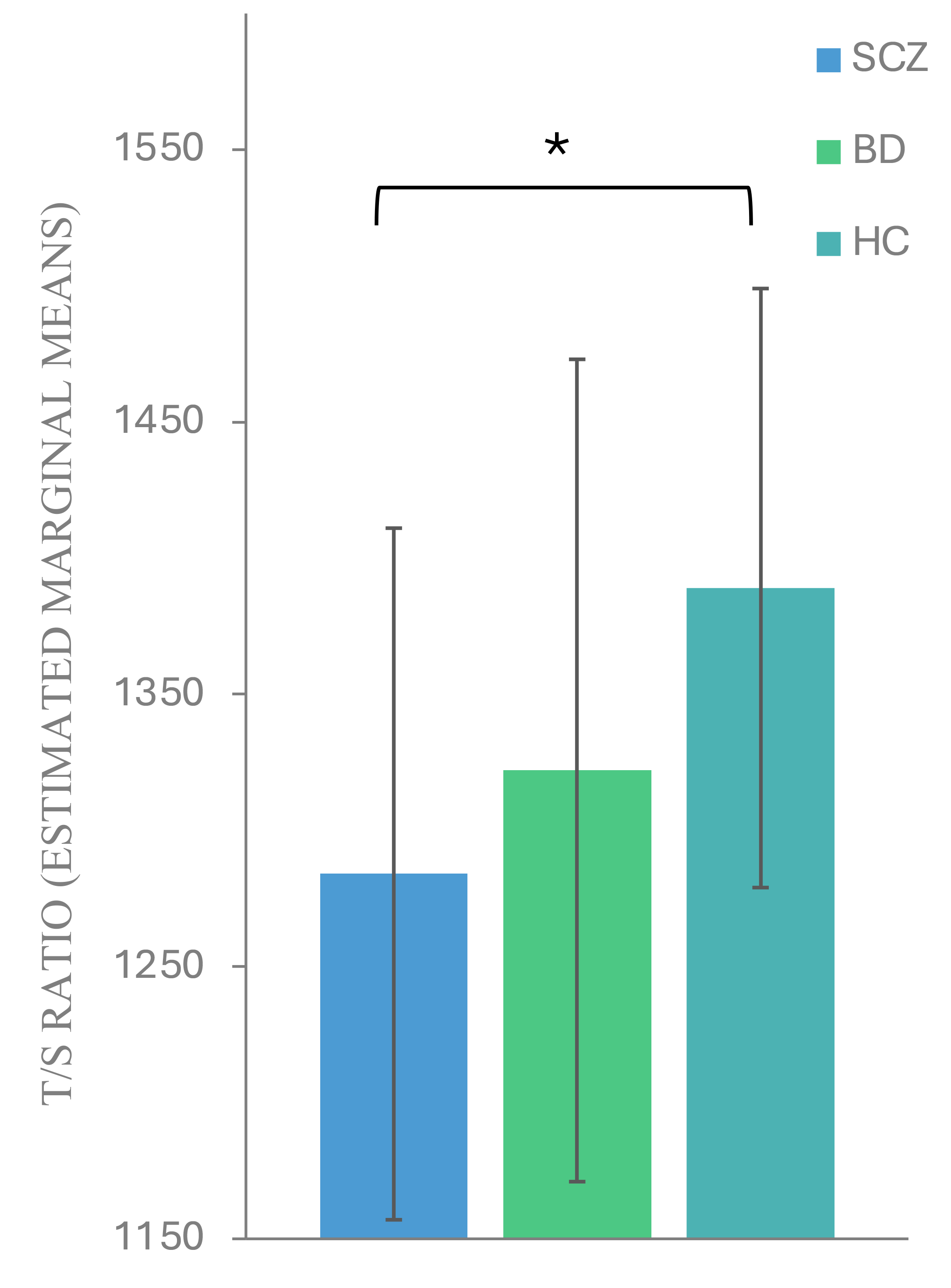


**Supplementary Figure 1** Difference in telomere length by T/S ratio among SCZ, BD and HC, presented by mean and confidence intervals based on estimated marginal means, including adjustments for age, sex, and BMI.

*Significant difference between SCZ and HC (*p* = 0.016).

Abbreviations: Bipolar disorder (BD), Body mass index (BMI), Healthy controls (HC), Schizophrenia spectrum disorders (SCZ), telomere template/amount of single-copy gene template (T/S ratio).
